# MicroRNA-20b and ERK1/2 pathway independently regulate the expression of tissue factor in hematopoietic and trophoblastic differentiation of human embryonic stem cells

**DOI:** 10.1186/scrt332

**Published:** 2013-10-11

**Authors:** Yan-Hui Yu, Deng-Shu Wu, Fang-Fang Huang, Zheng Zhang, Lin-Xin Liu, Jian Zhang, Hui-En Zhan, Min-Yuan Peng, Hui Zeng, Fang-Ping Chen

**Affiliations:** 1 Department of Hematology, Xiang-Ya Hospital, Central South University, 87 Xiang-ya Road, Changsha, Hunan 410008, China; 2 Department of Pharmacology, School of Pharmaceutical Sciences, Central South University, 85 Xiang-ya Road, Changsha, Hunan, China

## Abstract

**Introduction:**

Tissue factor (TF) is expressed in various types of cells. TF expression is essential for many biological processes, such as blood coagulation and embryonic development, while its high expression in stem cells often leads to failure of transplantation. In this study, we used the human embryonic stem cell (hESC) culture system to understand the molecular mechanisms by which TF expression is regulated in hESC-derived hematopoietic and trophoblastic cells.

**Methods:**

hESCs were induced *in vitro* to differentiate into hematopoietic and trophoblastic cells. TF expression in various types of cells during these differentiation processes was examined by quantitative real-time polymerase chain reaction analysis and western blot analysis. The regulatory mechanisms of TF expression were investigated by miRNA expression analysis, luciferase report assay, TF mRNA and protein analysis, and pathway phosphorylation analysis.

**Results:**

We first found that TF was expressed only in trophoblasts and granulocyte–monocyte (G-M) cells differentiated from hESCs; and then demonstrated that miR-20b downregulated and Erk1/2 signaling pathway upregulated the TF expression in trophoblasts and G-M cells. Finally, we found that miR-20b downregulated the TF expression independently of the Erk1/2 signaling pathway.

**Conclusions:**

The miR-20b and Erk1/2 pathway independently regulate expression of TF in trophoblasts and G-M cells differentiated from hESCs. These findings will open an avenue to further illustrate the functions of TF in various biological processes.

## Introduction

Tissue factor (TF) is a 47 kDa glycoprotein integrated in the membrane of cells [1]. As a receptor for factor II/FIIa, TF plays a pivotal role in extrinsic blood coagulation. Recently, emerging evidence has indicated its roles in tumor angiogenesis [2], inflammation, atherosclerosis [3,4], embryonic development [5], and homeostasis [6]. Much evidence has suggested that TF exerts pleiotropic roles in multiple biological processes via its varied expression in various types of cells. TF is widely expressed in many types of tissues with relatively high expression in the central nervous system, lungs, and placenta [7]. TF is also expressed in mature blood cells; however, its expression levels in blood cells are variable. For example, TF is highly expressed in granulocyte–monocyte (G-M) cells and macrophages [8,9], while its expression is rarely detectable in erythrocytes.

Varied TF expressions correspond to the functions of TF in some types of cells [10]. For example, in G-M cells, an essential component of the innate immune system, the expression of TF is increased when inflammation occurs. This observation reflects its role in blood coagulation and inflammation because inflammation activates the blood coagulation system and blood clotting activity in turn aggravates inflammatory reaction [11]. In this process, TF – a receptor molecule in G-M cells – activates the coagulation pathway and regulates inflammation reaction.

High expression of TF in granulocytes may cause graft-versus-host disease, a common complication that occurs in allogeneic cell and tissue transplantation. Graft-versus-host disease is characterized by immune complex formation, vascular rejection, activation of inflammation, vascular endothelial injury, and organ necrosis [12]. Increased TF expression in granulocytes provokes an immune response and then confers host body damage [13].

TF expression in the cells of the placenta is required for maintaining the stability of embryos [14]. The placenta is a highly vascularized organ with fetal and maternal blood supply. In the placenta, TF is only highly expressed in trophoblasts [15] that are essential for embryo implantation in and interaction with the decidualized maternal uterus [16]. This hemostatic balance may be critical for normal placental function and pregnancy outcome [17,18].

Although the expression of TF has been demonstrated in various biological processes, the molecular mechanisms regulating TF expression remains largely unknown. In recent years, microRNAs (miRNAs) have been found to participate in embryonic development by regulating gene expression [19]. miRNAs are small RNA molecules about 17 to 23 nucleotides in length. Usually, the miRNA binds to the miRNA–RNA-induced silencing complex in the cytoplasm, and this complex further binds to the 3′-untranslated region (UTR) of target transcripts and blocks protein translation or destabilizes mRNAs [20]. DNA analysis shows that there are miRNA-binding sites for miR-19a, miR-20b, and miR-106a in the 3′-UTR of the TF mRNA transcript. In human breast cancer cells, TF expression can be downregulated by miR-19 [21], suggesting that TF expression can be regulated by miRNA. Here, we hypothesized that the expression of TF in hematopoietic and trophoblastic cells differentiated from hESCs are regulated by miRNAs.

TF expression is also regulated by signaling pathways. In colorectal carcinoma cells, the activation of ras oncogene and inactivation of p53 leads to high expression levels of TF via the Mek1/2 and phosphatidylinositol 3-kinase pathway [22]. In lipoolysaccharide-stimulated human monocytic cells, the Erk1/2 specific inhibitor U0126 suppresses the TF promoter activity [23]. Furthermore, the Akt and Erk1/2 pathways have been shown to be involved in cellular development and cell proliferation [24]. In this study, we also asked whether Akt or Erk1/2 participates in regulating TF expression.

Human embryonic stem cells (hESCs) can be successfully expanded and induced to differentiate into all stages of hematopoietic cells and trophoblasts *in vitro*. In this study, we used this system to address the following questions: is TF expressed in various types of cells during these differentiation processes? Are miRNAs, the Erk1/2 signaling pathway or the Akt signaling pathway involved in the regulation of TF expression?

## Materials and methods

### Cell cultures and differentiation

The hESC lines H9 and CT2 were maintained in the presence of 4 ng/ml basic fibroblast growth factor (R&D Systems, Minneapolis, MN, USA) as described previously [25]. Trophoblastic differentiation of hESCs was carried out in medium with 100 ng/ml BMP-4 (R&D Systems) for up to 5 to 7 days as described elsewhere [26]. Hematopoietic differentiation of hESCs was carried out as described previously [27]. Briefly, hESCs were transferred onto OP9 feeders and cultured in α-mimimum essential medium (MEM) supplemented with 10% fetal bovine serum, 2 mM l-glutamine, 10% Nonessential Amino Acids (NEAA), and 1-thioglycerol for 7 days to allow hESCs to differentiate into hematopoietic stem/progenitor cells (HSPCs) (CD34^+^CD38^–^). On day 8, HSPCs were selected by magnetic activated cell sorting and further differentiated into either G-M cells (CD14^+^CD34^–^ or CD15^+^CD34^–^) by culturing them in the medium supplemented with G-CSF (100 ng/ml; R&D Systems) for 7 days or into erythrocytes (CD235^+^) in medium supplemented with EPO (100 ng/ml; R&D Systems) for 14 days. The G-M cells were maintained in Dulbecco’s modified Eagle’s medium–F12 with 10% fetal bovine serum and interleukin-3 (50 ng/ml; R&D Systems). Erythrocytes were maintained in Dulbecco’s modified Eagle’s medium–F12 with 30% fetal bovine serum and IL-3 (50 ng/ml; R&D Systems). The Ethics Committee of Xiangya Hospital of Centre South University approved the study.

### Florescence-activated flow cytometry

Surface markers of cells were analyzed using florescence-activated flow cytometry (FACS). Cells were stained with various combinations of monoclonal antibodies conjugated with fluorochromes. Antibodies, CD14-phycoerythrin (PE), CD15-allophycocyanin (a surface marker for G-M cells), CD34-PE (a surface marker for HSPCs), CD235a-PE (a surface marker for erythrocytes), and CD142-fluorescein isothiocyanate (TF) were purchased from BD Biosciences (BD, San Jose, CA, USA). Stained cells were analyzed using a FACS Calibur flow cytometer (BD Biosciences) and the data were analyzed with FlowJo software (Milteneyi Biotech, Auburn, CA, USA).

### Magnetic activated cell sorting

To isolate CD34^+^CD38^–^, CD14^+^CD34^–^, or CD15^+^CD34^–^ hematopoietic cells from cultured cells, we used a MACS Pro Separator (Milteneyi Biotech, Auburn, CA, USA). Dead cells in the culture were excluded by staining with 7-aminoactinomycin staining solution (BD Biosciences) and live cells were stained with CD14-PE, CD15-allophycocyanin, CD34-PE, or CD235a-PE before separation. TF expression in these purified hematopoietic cell populations was evaluated by FACS after staining cells with CD142-fluorescein isothiocyanate antibody.

### Plasmid construction

**Figure 1:**
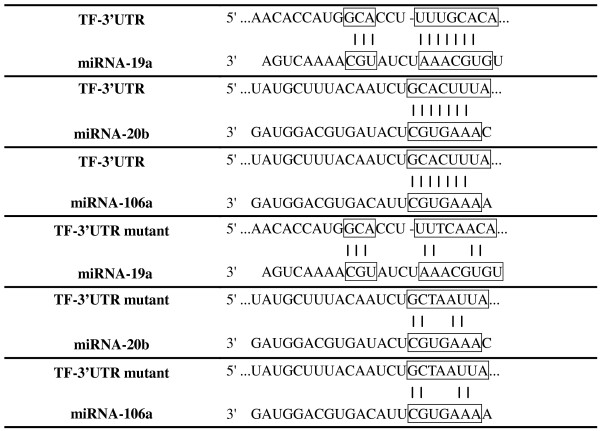
**Potential microRNA binding sites in the 3′-untranslated region of the tissue factor gene.** miRNA, microRNA; TF, tissue factor; UTR, untranslated region.

To construct the dual-luciferase vector, pmirGLO-TF-3′-UTR bearing the luciferase reporter gene with the 3′-UTR of TF in the promoter region, a 1,200 base pair fragment (NM_001993, 1141 to 2,341 base pairs) was first amplified using polymerase chain reaction (PCR) with the forward primer 5′-ATAGAGCTCAGGAAGCACTGTTGGAGC-3′ (27 base pairs) and the reverse primer 5′-TAAGTCGACGCGAAAAAGATACGTTGTTG-3′ (29 base pairs). The amplified fragment was then cloned into the pmirGLO vector (Promega, Madison, WI, USA) (Figure 1). The pmirGLO-TF-3′-UTR mutant was constructed by cloning the TF-3′-UTR mutant fragment, which was generated using the site-directed mutagenesis kit (Stratagene, La Jolla, CA, USA).

### Cell transfection

The pmirGLO-TF-3′-UTR and its corresponding mutant plasmid DNA were prepared as usual. miRNA mimics and inhibitors for miR-19a, miR-20b, and miR-106a were purchased from GenePharma Co. (Shanghai, China). For transfection, G-M cells were cultured in a flask at a cell density of 10^7^/ml and trophoblasts were plated in plates at 80% confluence. Twenty-four hours later, these cells were washed twice with Dulbecco’s phosphate-buffered saline (DPBS, SIGMA, St. Louis, Missouri, USA) and then transfected with 2 μg TF-3′-UTR or mutant plasmid DNA with 100 nM inhibitors or 100 nM mimics of miR-19a, miR-20b, or miR-106a mixed with Lipofectamine 2000 (Invitrogen, Carlsbad, CA, USA) according to the manufacturer’s instructions. The transfection procedure was repeated twice at 24 hours and 48 hours following the first transfection. Randomly synthesized RNA fragments were used as control. After 3 days, cells were washed twice in Dulbecco’s phosphate-buffered saline, filtered through a 70 μm cell strainer (BD Biosciences), and used for further analysis.

### Luciferase assay

Luciferase activity in cells was assayed using the Luciferase Assay Kit (Promega) according to the manufacturer’s instructions. Briefly, one million cells were transfected, harvested, and lysed at 48 hours after cell transfection. Then 20 μl cell lysate was mixed with 100 μl Luciferase Assay Reagent. Light produced was measured using a BMG FLUOstar Optima (BMG Labtech GmbH, Germany).

### Inhibition of Erk1/2 signaling pathway

To inhibit the Erk1/2, G-M cells or trophoblasts were cultured in differentiation medium in the presence of 10 μM U0126 (Cell Signal Technology, Danvers, MA, USA) for 48 hours.

#### ***Semiquantitative reverse transcription-PCR***

Total RNA was extracted by Trizol reagent (Invitrogen) and reverse transcribed to cDNA using the SuperScript^®^ RT Kit (Invitrogen) according to the manufacturer’s instructions. Primers used for semiquantitative reverse transcription-PCR to measure expression of TF, CDX2, Oct-4, and Nanog are presented in Table 1. PCR was carried out in GeneAmp 9700 (Applied Biosystems, Foster City, CA, USA) with the following PCR programs: TF – 95°C for 5 minutes; 32 cycles of 94°C for 30 seconds, 50°C for 30 seconds, and 72°C for 30 seconds; and 72°C for 10 minutes; and CDX2, Oct-4, and Nanog – 95°C for 5 minutes; 32 cycles of 94°C for 30 seconds, 62°C for 30 seconds, and 72°C for 30 seconds; and 72°C for 10 minutes.

**Table 1:** P**rimer pairs used for reverse transcriptase polymerase chain reaction**

**Gene symbol**	**Forward primer (5′ to 3′)**	**Reverse primer (5′ to 3′)**
Oct-4	CTTGGGCTACACAGGC	CTCAATACTCGTTCGCTTTC
Nanog	TTTGGAAGCTGCTGGGGAAG	GATGGGAGGAGGGGAGAGGA
CDX2	CCGAACAGGGACTTGTTTAGAG	CTCTGGCTTGGATGTTACACAG
TF	ACGCTCCTGCTCGGCTGGGT	CGTCTGCTTCACATCCTTCA
GAPDH	GGAGCCAAAAGGGTCATC	CCAGTGAGTTTCCCGTTC

### Quantitative real-time PCR

Total RNA including small RNAs was isolated from cultured cells using the miRNA-RT Kit (Takara, Dalian, China) according to the manufacturer’s instructions. miRNAs were quantified by quantitative real-time PCR using the SYBR mix (Takara) and the primers presented in Table 2 according to the manufacturer’s instructions. PCR was carried out in 7900HT (Applied Biosystems).

**Table 2:** Primer pairs used for quantitative real-time polymerase chain reaction

**Gene symbol**	**Forward primer (5′ to 3′)**	**Reverse primer (5′ to 3′)**
PU.1	CCTGTATGTAGCGCAAGAGATTTA	CCAGCACAAGTTCCTGATTTTATC
CDX2	AGGGGGTGGTTATTGGACTC	CATTCAGCCCAGAGAAGCTC
TF	GCCAGGAGAAAGGGGAAT	CAGTGCAATATAGCATTTG
GAPDH	ACAGTCAGCCGCATCTTCTT	ACGACCAAATCCGTTGACTC

### Western blotting

Total proteins in cultured cells were prepared by lysing cells in RIPA buffer with protease inhibitors (Sigma-Aldrich). Equal amounts of protein were separated on a 10% SDS polyacrylamide gel and then transferred onto a polyvinylidene fluoride membrane (Millipore, Billerica, MA, USA). After blocking with 0.5% bovine serum albumin (Abcam, Cambridge, MA, USA), the polyvinylidene fluoride membranes were incubated for 1 to 2 hours at room temperature with TBST-diluted primary antibodies against TF (1:200; Abcam), Erk1/2 (1:500; Cell Signal Technology), phosphorylated Erk1/2 (1:500; Cell Signal Technology), Akt (1:500; Cell Signal Technology), phosphorylated Akt (1:500; Cell Signal Technology) or β-Actin (1:1,000; Cell Signal Technology) followed by incubation with horseradish peroxidase-linked secondary antibody (1:2,000; Santa Cruz Biotechnology, Inc., Santa Cruz, CA, USA) for 1 hour at room temperature. Finally, the membranes were visualized by the Che-mi Doc imaging system (Bio-rad, Hercules, CA, USA) or Immobilon Western Chemiluminescent HRP Substrate (Millipore).

### Statistical analysis

All experiments were repeated at least three times. In each experiment, triplicate samples were used to analyze for each parameter described above. All values were expressed as means ± standard error of the mean. *P* <0.05 was considered statistically significant. Statistical analysis was performed using SPSS software (version 17.0; Millipore, Billerica, MA, USA).

## Results

### Expression of TF in trophoblasts and hematopoietic cells differentiated from hESCs

**Figure 2:**
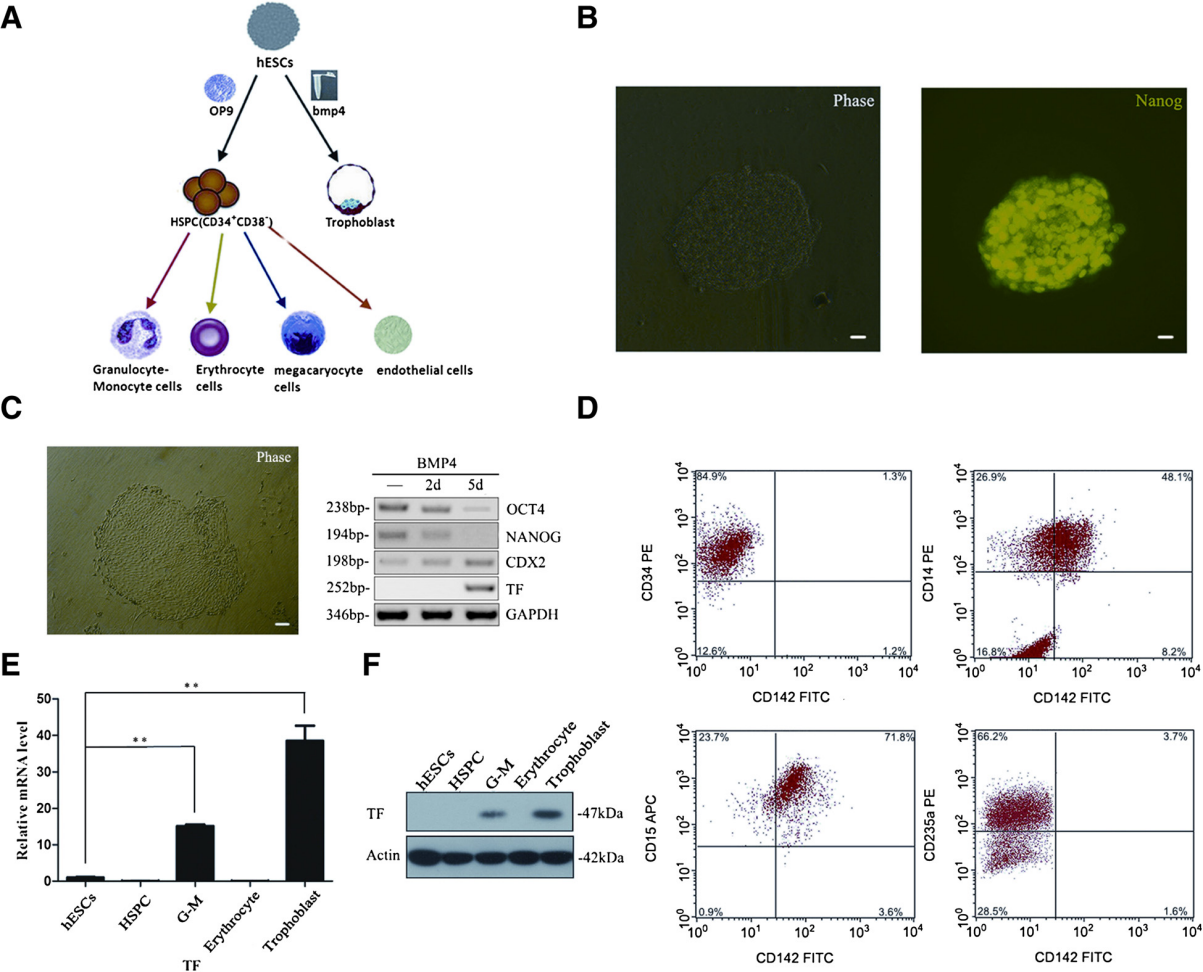
**Tissue factor differential expression in trophoblasts and hematopoietic cells derived from human embryonic stem cells. (A)** Schematic diagram of human embryonic stem cell (hESC) differentiation into hematopoietic and trophoblastic cells. **(B)** Immunostaining showed hESCs expressing Nanog. **(C)** Trophoblastic differentiation of H9 cells. H9 hESCs were induced in medium with BMP4 to differentiate into trophoblasts. Expression of Oct-4, Nanog, CDX2, and tissue factor (TF) were examined using reverse-transcription polymerase chain reaction (PCR). **(D)** H9 hESCs were induced to differentiate into hematopoietic cells. Florescence-activated flow cytometry (FACS) analysis showed that TF (CD142) was expressed in CD14^+^ and CD15^+^ G-M cells, but not in CD34^+^ hematopoietic stem/progenitor cells (HSPCs) and CD235^+^ erythrocytes. **(E)** TF mRNA in different types of hematopoietic cells was examined by quantitative real-time PCR. **(F)** TF protein in different types of hematopoetic cells and trophoblasts was examined by western blotting. APC, allophycocyanin; bp, base pair; FITC, fluorescein isothiocyanate; GAPDH, glyceraldehyde 3-phosphate dehydrogenase; G-M, granulocyte–macrophage; PE, phycoerythrin.

*In vitro*, H9 and CT2 hESCs were successfully induced to differentiate to trophoblasts and HSPCs, and then G-M cells and erythrocytes (Figure 2A). Proliferating H9 hESCs expressed Nanog, Oct4, and a low level of CDX2 (Figure 2B,C). The expression of Oct4 and Nanog began to decrease at differentiation day 2 and almost disappeared at differentiation day 5 toward trophoblasts while the expression of CDX2, a trophoblast marker gene, increased with time (Figure 2C). These results indicated that induced differentiation toward trophoblasts was successful. We then asked whether TF was expressed in trophoblasts by reverse transcriptase PCR and western blotting. As shown in Figure 2C,F, TF was not expressed in proliferating embryonic stem cells and cells at differentiation day 2, but was expressed in cells at differentiation day 5.

We purified HSPCs, G-M cells, and erythrocytes and examined the expression of TF in these cells by FACS analysis, quantitative real-time PCR, and western blotting. Only G-M cells, including CD14^+^ and CD15^+^ cells, expressed CD142 (TF) (Figure 2D,E,F). Likewise, TF was only expressed in the trophoblasts and G-M cells, but not in HSPCs and erythrocytes differentiated from CT2 hESCs (data not shown). Taken together, these results suggested that TF was expressed only in G-M cells and trophoblasts differentiated from hESCs.

### miR-20b inhibited TF expression in trophoblasts, and G-M cells differentiated from hESCs

**Figure 3:**
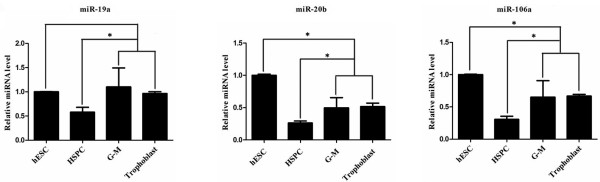
**miR-19a, miR-20b, and miR-106a expression in hematopoietic cells and trophoblasts derived from human embryonic stem cells.** Total RNAs from human embryonic stem cells (hESCs), hematopoietic stem/progenitor cells (HSPCs), granulocyte–macrophage (G-M) cells, and trophoblasts were extracted and the expression of miRNAs was analyzed by quantitative real-time polymerase chain reaction. *P <0.05.

In the 3′-UTR of TF mRNA, there are binding sites for miR-19a, miR-20b, and miR-106a (Figure 1). We thus asked whether these miRNAs regulated TF expression by examining their expression patterns in hESCs, trophoblasts, HSPCs, and G-M cells. The expression pattern of any miRNA corresponding to the TF expression pattern would suggest its potential regulatory role. Surprisingly, the expressions of miR-20b and miR-106a were significantly higher in hESCs than in HSPCs, G-M cells, and trophoblasts. The expression of all three miRNAs in HSPCs was significantly lower than in G-M cells and trophoblasts (Figure 3). These miRNA expression patterns were also observed in the cells differentiated from CT2 hESCs (data not shown).

**Figure 4:**
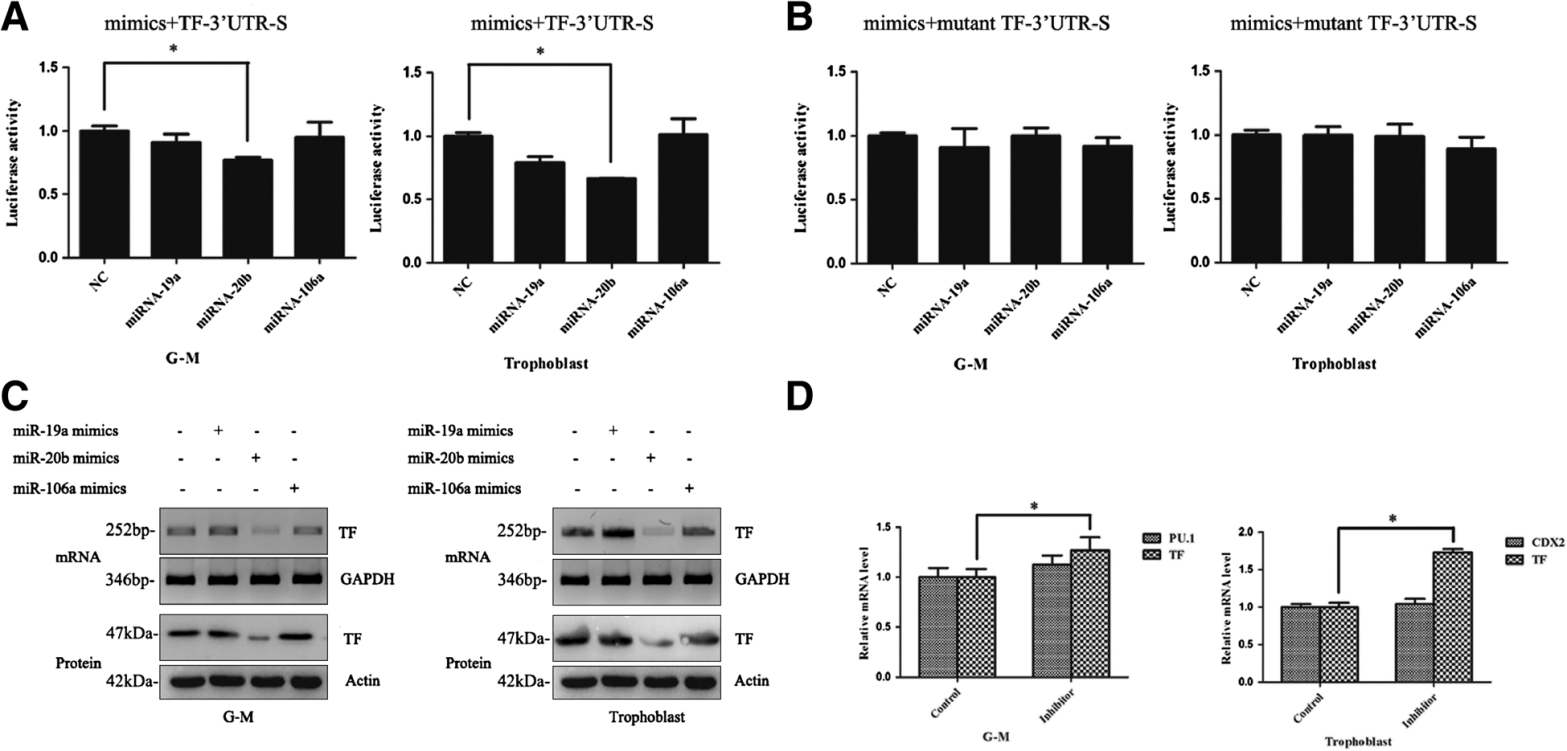
**miR-20b inhibited tissue factor expression. (A), (B)** Tissue factor (TF)-3′-untranslated region (UTR)-luciferase reporter assay. Various miRNA mimics with either **(A)** wild-type or **(B)** mutant TF-3′-UTR-luciferase reporter gene were transfected into granulocyte–macrophage (G-M) cells and trophoblasts, respectively. Then 48 hours post transfection, luciferase activity was measured and reported as the mean ± standard error of the percentages of the luciferase activity in cells without miRNA mimics. **(C)**, mRNA and protein analysis in G-M cells and trophoblasts transfected with various miRNA mimics. **(D)** TF, PU.1, and CDX2 mRNA levels in G-M cells and trophoblasts transfected with miR-20b inhibitor were analyzed using quantitative real-time polymerase chain reaction and reported as the mean ± standard error of the percentage of the mRNA levels of their corresponding gene in control cells, respectively. *P <0.05. bp, base pair; NC.

We therefore asked whether miR-19a, miR-20b or miR-106a mimics could alter TF expression in G-M cells and trophoblasts using the TF-3′-UTR reporter assay, TF mRNA, and TF protein analysis. In the TF-3′-UTR reporter assay, only miR-20b mimics significantly decreased the reporter activity in both G-M cells and trophoblasts (Figure 4A). The suppression of miR-20b on TF-3′-UTR reporter was specific because miR-20b mimics could not inhibit the reporter activity driven by mutant TF-3′-UTR (Figure 4B). Similarly, reverse transcriptase PCR for TF mRNA and western blotting for TF protein also showed that TF expression in G-M cells or trophoblasts was reduced by miR-20b mimics, but not by miR-19a or miR-106a mimics (Figure 4C).

To further confirm our observation above, we asked whether miR-20b inhibitor could increase the TF expression in G-M cells or trophoblasts. As shown in Figure 4D, TF mRNA was significantly increased in both trophoblasts and G-M cells when miR-20b inhibitor was administrated, while this administration did not affect the expression of the lineage-specific marker PU.1 in G-M cells or CDX2 in trophoblasts. These results were also observed in the cells differentiated from the CT2 hESCs (data not shown).

Taken together, these data suggested that miR-20b decreased TF expression, while it did not disturb the trophoblastic or hematopoietic differentiation of hESCs.

### Erk1/2 pathway is involved in regulating TF expression in trophoblasts and G-M cells differentiated from hESCs

**Figure 5:**
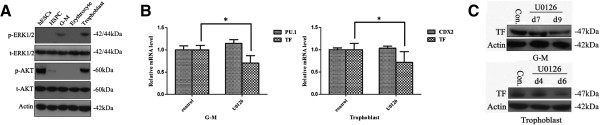
**Erk1/2 signaling pathway involved in regulating tissue factor expression in trophoblastic and hematopoietic differentiation of human embryonic stem cells. (A)** Western blot analysis of phosphorylated Erk1/2 and Akt in various types of cells showing that Erk1/2 signaling pathway is active in granulocyte–macrophage (G-M) cells and trophoblasts. p-Erk1/2, phosphorylated Erk1/2; t-Erk1/2, total Erk1/2; pAkt, phosphorylated Akt; t-Akt, total Akt. **(B),(C)** Decreased **(B)** mRNA and **(C)** protein levels of tissue factor (TF) in G-M cells or trophoblasts treated with Erk1/2-specific inhibitor, U0126. Cells were treated with 10 μM U0126 or dimethylsulfoxide (control) for 4-6 or 7-9 days before harvest for quantitative real-time polymerase chain reaction for CDX2, PU.1, and TF mRNA levels. Data were reported as the mean ± standard error of the percentage of the mRNA levels of CDX2, PU.1, and TF in cells from the control group. *P <0.05. Western blotting analysis was carried out on the days designated in **(C)**. hESC, human embryonic stem cell; HSPC, hematopoietic stem/progenitor cell.

TF has been reported to be a target gene of Akt and Erk1/2 pathways in human umbilical vein endothelial cells and breast cancer cells [28,29]. We asked whether these pathways were involved in regulating TF expression in the trophoblasts and hematopoietic cells differentiated from hESCs. We first asked whether the Erk1/2 or Akt signaling pathway was activated in hESCs, HSPCs, G-M cells, erythrocytes, and trophoblasts by examining the levels of phosphorylated Erk1/2 or Akt. Phosphorylated Erk1/2 was detected in trophoblasts and G-M cells, but not in hESCs, HSPCs, and erythrocytes, while phosphorylated Akt was detected in hESCs and trophoblasts, but not in HSPCs, G-M cells, and erythrocytes (Figure 5A). The Erk1/2 pathway activity thus corresponded to TF expression in G-M cells and trophoblasts.

To confirm this observation, we used U0126 to specifically inhibit Erk1/2 pathway activity and asked whether this treatment altered the expression of TF, PU.1 (G-M cell-specific marker gene), and CDX2 (trophoblast-specific marker gene) in G-M cells and trophoblasts. We found that inhibiting the Erk1/2 signaling pathway significantly reduced the levels of mRNA (Figure 5B) and protein (Figure 5C) of TF in both G-M cells and trophoblasts. Interestingly, inhibiting Erk1/2 pathway activity did not alter the mRNA levels of PU.1 in G-M cells and CDX2 in trophoblasts (Figure 5B). Likewise, we also found that inhibiting the Erk1/2 signaling pathway using U0126 significantly reduced the expression of TF in both G-M cells and trophoblasts differentiated from CT2 hESCs (data not shown). Taken together, these results suggested that Erk1/2 pathway upregulated TF expression in G-M cells and trophoblasts.

### miR-20b downregulated TF expression in G-M cells and trophoblasts but not through the Erk1/2 pathway

**Figure 6:**
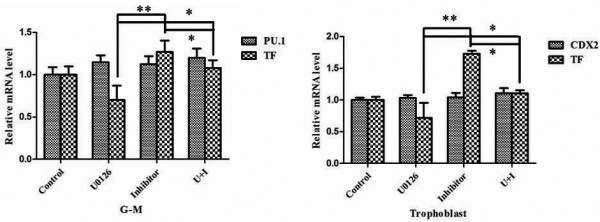
**Erk1/2 signaling pathway and miR-20b regulate tissue factor expression in trophoblastic and hematopoietic differentiation of human embryonic stem cells.** Granulocyte–macrophage (G-M) cells and trophoblasts were treated with both miR-20b inhibitor and Erk1/2-specific inhibitor U0126, simultaneously for 48 hours before harvesting for quantitative real-time polymerase chain reaction to measure the mRNA levels of PU.1, CDX2, and tissue factor (TF). Data reported as the mean ± standard error of the percentage of the mRNA levels of the corresponding gene in cells without treatment. *P <0.05, **P <0.01.

Both miR-20b and the Erk1/2 signaling pathway regulated TF expression in G-M cells and trophoblasts. miR-20b may regulate the expression of other genes related with Erk1/2 signaling pathway activity. We thus asked whether miR-20b inhibited TF expression via the Erk1/2 signaling pathway in these cells. For this purpose, we asked whether specifically blocking Erk1/2 pathway activity using U0126 could prevent the upregulated TF mRNA levels using miR-20b inhibitor. As shown in Figure 6, administration of U0126 only partially reduced the upregulated mRNA levels of TF in G-M cells and trophoblasts using miR-20b inhibitor. Likewise, the same results were also observed in the G-M cells and trophoblasts differentiated from CT2 hESCs (data not shown). These data suggest that miR-20b did not regulate TF expression through the Erk1/2 signaling pathway.

## Discussion

To understand the molecular mechanisms by which TF differential expression was regulated, we used a hESC culture system that allows us to mimic the hematopoietic and trophoblastic developmental processes. In this system, we demonstrated that TF was expressed only in G-M cells and trophoblasts (Figure 2), consistent with the previous observation that TF expression is regulated in cells to exert its functions in various biological processes.

Because bioinformatic analysis of the 3′-UTR of the TF transcript suggests that TF expression may be regulated by miR-19a, miR-20b, and miR-106a, we investigated the potential of these miRNAs to regulate TF expression in G-M cells and trophoblasts differentiated from hESCs and found that miR-20b mimics inhibited TF expression in these cells, but did not disturb the differentiation process because the expression of G-M cell-specific marker gene PU.1 or the trophoblast-specific marker gene CDX2 was not affected (Figure 4). Our conclusion is based on the following results: all three miRNAs had lower expression levels in all hematopoietic cells and trophoblasts differentiated from hESCs than their parent hESCs (Figure 3); only miR-20b mimics specifically decreased the activity of the TF-3′-UTR-driven luciferase reporter, but not the mutant TF-3′-UTR-driven reporter (Figure 4A,B) when they were analyzed in G-M cells or trophoblasts; only miR-20b mimics inhibited the TF expression in G-M cells and trophoblasts (Figure 4C); and miR-20b inhibitor increased the TF expression in G-M cells and trophoblasts (Figure 4D). Several studies have shown that many types of cancer cells express aberrantly high levels of TF [22] and miR-19 regulates TF expression in breast cancer cells [30]. We here provided evidence showing that miR-20b may directly interact with the 3′-UTR of TF to suppress the expression of TF. In contrast, HSPCs had the lowest levels of miR-20b among hESCs, G-M cells, and trophoblasts, but did not express TF (Figure 3). Therefore, it is very possible that TF expression is also regulated by other mechanisms.

Our study did conclude that the Erk1/2 signaling pathway regulated the TF expression independent of miR-20b. First, phosphorylated Erk1/2 was detected in G-M cells and trophoblasts, but not in hESCs and HSPCs (Figure 5A). Second, specifically inhibiting the Erk1/2 signaling pathway decreased TF expression in G-M cells and trophoblasts (Figure 5B,C). Erk1/2-regulated or Akt-regulated TF expression is also observed in endothelial and breast cancer cells [28,31]. Inhibiting Erk1/2 pathway activity did not block the upregulation of TF expression conveyed by introducing miR-20b inhibitor in G-M cells and trophoblasts (Figure 6).

Interestingly, our data showed that introducing miR-20b inhibitor to increase the TF expression or inhibiting Erk1/2 pathway activity to decrease TF expression, or both, did not disturb the hematopoietic and trophoblastic differentiation of hESCs because either treatment to G-M cells or trophoblasts did not alter the G-M cell-specific marker PU.1 and the trophoblast-specific marker CDX2 (Figure 6). This result implicated that TF expression may not be related to hematopoietic or trophoblastic differentiation of hESCs.

## Conclusions

In summary, we successfully used the hESC culture system to investigate the molecular mechanisms by which TF expression in hematopoietic and trophoblastic differentiation of hESCs is regulated. We found that miR-20b downregulated and the Erk1/2 signaling pathway upregulated TF expression in G-M cells and trophoblasts differentiated from hESCs. Both the miRNA and the Erk1/2 pathway regulated TF expression in these cells independently and did not affect the hematopoietic and trophoblastic differentiation of hESCs. Our study initiates a way to illustrate the cellular functions of differential expression of TF.

## Abbreviations

Erk1/2: Extracellular signal-regulated kinase; FACS: Florescence-activated flow cytometry; G-M: Granulocyte–monocyte; hESC: Human embryonic stem cell; HSPC: Hematopoietic stem/progenitor cell; miRNA: microRNA; PCR: Polymerase chain reaction; PE: Phycoerythrin; TF: Tissue factor; UTR: Untranslated region.

## Competing interests

The authors declare that they have no competing interests.

## Authors’ contributions

HZ and F-PC were responsible for the concept of the study. Y-HY, HZ and F-PC designed the study. Y-HY, D-SW, ZZ, F-FH and M-YP carried out the experiments. Y-HY, H-EZ, L-XL and JZ performed the statistical analyses. Y-HY and HZ wrote the manuscript. HZ and F-PC approved the final version of the manuscript. All authors read and approved the final manuscript.
